# Serum SHARP1 and uterine artery Doppler for the prediction of preeclampsia

**DOI:** 10.1038/s41598-019-48727-8

**Published:** 2019-08-22

**Authors:** Noppakorn Prakansamut, Vorapong Phupong

**Affiliations:** Department of Obstetrics and Gynecology, Faculty of Medicine, Chulalongkorn University, Rama IV Road, Pathumwan, Bangkok 10330 Thailand

**Keywords:** Predictive markers, Endocrine reproductive disorders

## Abstract

The aim of this study was to identify the value of serum SHARP1 levels and Doppler of the uterine artery in singleton pregnancy at 11–13^+6^ weeks for predicting preeclampsia. A prospective observational study was conducted in pregnant women at 11–13^+6^ weeks of pregnancy who had antenatal care at King Chulalongkorn Memorial Hospital, Chulalongkorn University, Bangkok, Thailand, between January 2017 and January 2018. Serum SHARP1 measurement and transabdominal Doppler of the uterine artery were performed. The predictive values of these tests were determined. Data were obtained from 405 pregnant women. Thirty-five women had preeclampsia (8.6%), and six of these had early-onset preeclampsia (1.5%). Preeclamptic women had significantly lower serum SHARP1 levels than pregnant women without preeclampsia (3.6 ng/ml vs 4.7 ng/ml, p < 0.01). The sensitivity, specificity, positive predictive value (PPV) and negative predictive value (NPV) of serum SHARP1 levels of less than 3.89 ng/ml for predicting preeclampsia were 77.1%, 72.7%, 21.1% and 97.1%, respectively. For uterine artery Doppler, the sensitivity, specificity, PPV and NPV of the mean pulsatility index (PI) > 95^th^ percentile for predicting preeclampsia were 5.7%, 95.4%, 10.5% and 91.5%, respectively. For the combination of serum SHARP1 levels with a cutoff value of less than 3.89 ng/ml and a mean PI > 95^th^ percentile, the sensitivity, specificity, PPV and NPV were 77.1%, 70.3%, 19.7% and 97.0%, respectively. This study demonstrated that serum SHARP1 is a promising biomarker for predicting preeclampsia in the first trimester.

## Introduction

Preeclampsia is a multisystem disorder characterized by new-onset hypertension and proteinuria after 20 weeks of gestation. This disorder causes serious complications during pregnancy, such as acute renal failure, pulmonary edema and coagulopathy. Preeclampsia is one of the most common causes of maternal morbidity and mortality worldwide^1,2^. Moreover, it is associated with increased risk of perinatal morbidity and mortality and of long-term maternal health consequences, e.g., cardiovascular disease^3^. Preeclampsia occurs in approximately 2–8% of all pregnancies depending on race, environment and diagnostic criteria^4–6^. At King Chulalongkorn Memorial Hospital, the incidence of preeclampsia was 5.7% in 2015^7^.

Screening for preeclampsia in the first trimester aims for early identification of women at high risk of developing preeclampsia who would potentially benefit from prophylactic pharmacological interventions, such as aspirin^4^, and appropriate antenatal care^4,8^. Effective screening tests for preeclampsia have not yet been identified. The 2015 guidelines of the American College of Obstetricians and Gynecologists^9^ recommend only an appropriate medical history to evaluate maternal risk factors as a screening tool to predict preeclampsia, but the detection rate is only 30%^10^.

To date, numerous studies have focused on predictive tests for preeclampsia to identify a more effective and accurate test with higher sensitivity and specificity than maternal risk factors alone. Using combined multiple predictors to predict preeclampsia can improve both the sensitivity and specificity over that of a single predictor^10–13^.

Split and Hairy-related Protein 1(SHARP1) is a member of the transcriptional repressor subfamily of the basic helix-loop-helix super family (bHLH) that is expressed in various embryonic and adult tissues. The SHARP1 gene is approximately 5 kbp in length and contains 5 exons. SHARP1 plays a role in the adaptation to environmental signal, especially oxygen concentrations^14^.

Hypoxia- inducible factor-1 (HIF-1) is a primary transcriptional mediator of the hypoxic response and master regulator of oxygen homeostasis. HIF-1 is expressed when a balance between the oxygen supply and usage in tissues cannot be attained^15^. Under hypoxic conditions, HIF-1 activates the transcription of genes encoding proteins that mediate adaptive responses to reduced oxygen availability. Concurrently, SHARP1 mRNA expression is also induced. A SHARP1 mediated feedback loop participates in the regulation of gene expression induced by hypoxia, such as VEGF mRNA expression. SHARP1 functions as a negative regulator of VEGF mRNA that has been induced by HIF-1 under hypoxic conditions^15–17^. VEGF mRNA plays an important role in angiogenesis. One study found that SHARP1 levels decreased in cases of preeclampsia^18^.

No study has evaluated the value of serum SHARP1 levels in the first trimester for predicting preeclampsia. Thus, the objective of this study was to find the value of serum SHARP1 levels and Doppler of the uterine artery in the first trimester for predicting preeclampsia.

## Materials and Methods

This prospective observational study was conducted in the Department of OB&GYN, King Chulalongkorn Memorial Hospital, Faculty of Medicine, Chulalongkorn University, Bangkok, Thailand between January 2017 and January 2018. The study was approved by the Institutional Review Board. All procedures were performed in accordance with the relevant guidelines and regulations of the Institutional Review Board. All subjects gave written informed consent.

Women with singleton pregnancy and a gestational age (GA) of 11–13^+^^6^ weeks were invited into the study. GA was calculated from the last menstrual period and documented by first trimester ultrasonogram. The exclusion criteria included the use of aspirin or any anticoagulant drug by the pregnant women and the presence of fetal abnormalities.

The sample size calculation was based on the sensitivity for predicting preeclampsia from Ersoy AO, *et al*’s study (67%)^18^. Twenty-one preeclamptic women were needed to detect significant difference (α = 0.05, allowable error  = 0.2). Considering the incidence of preeclampsia at our institute of 5.7%, 405 women were required for this study, with adjustments for a loss to follow-up rate of 10%.

The objective was to find the value of serum SHARP1 levels and Doppler of the uterine artery for predicting preeclampsia. Preeclampsia was diagnosed by an increased blood pressure of at least 140/90 mmHg measured on 2 occasions at least 6 hours apart, with proteinuria evidenced by a reading of at least 1 + on the urine dipstick test, or a protein excretion rate of at least 300 mg/24 hours, or a urine protein/creatinine ratio (UPCI) of >0.3. Both increased blood pressure and proteinuria occurred for the first time later than 20 weeks of pregnancy^19^. Early-onset preeclampsia was defined as preeclampsia occurring at a GA of less than 34 weeks. Late-onset preeclampsia was defined as preeclampsia occurring at a GA of 34 or more weeks^20^.

Maternal demographic data, the serum SHARP1 level, Doppler of the uterine artery pulsatility index (PI), and maternal and neonatal outcomes were obtained.

### Doppler of the uterine artery study

Flow velocity waveforms of the uterine artery were performed by ultrasound machines using an AB 2–7 MHz convex abdominal probe (GE Voluson E10, GE Medical Systems, Zipf, Austria). Each woman was evaluated once in the semirecumbent position by a single operator after bed rest for 5 minutes. The technique used for Doppler of uterine artery measurement was previously described^21,22^. Three consecutive waveforms were obtained in the Doppler study. The mean PI was calculated. The presence or absence of an early diastolic notch was documented. An early diastolic notch was documented by a definite upward change in velocity after the deceleration slope of the primary wave. An abnormal Doppler of uterine artery result was diagnosed as a mean PI> the 95th percentile for each GA.

### Sample collection for serum SHARP1 measurement

After the Doppler study was performed, blood was drawn. Blood was collected into nonheparinized tubes. Blood samples were centrifuged for 10 minutes at 2,500 rpm and stored until assayed at −80 °C. Maternal serum SHARP1 levels were measured by enzyme-linked immunosorbent assays (ELISA) (Cloud-Clone Corp, Massachusetts, TX, USA) according to the company instruction. This kit is a sandwich enzyme immunoassay kit. In the assay, controls, standards, and serum samples are incubated in microtitration wells that have been coated with a biotin-conjugated antibody against SHARP1. Detection reagent A is added to the wells after incubation and liquid removal. The wells are incubated with detection reagent B after a second incubation and wash step. The wells are incubated with substrate solution after a third incubation and wash step. A stop solution is then added after a fourth incubation. The degree of enzymatic turnover of the substrate is measured by using a microplate reader to measure the absorbance at a wavelength of 450 nm. The minimum detectable SHARP1 concentration in the assay was 0.156 ng/ml, as reported by the manufacturer. The intra-assay and inter-assay variations were less than 10%.

### Statistical analysis

Data analyses were performed by using the SPSS software package version 22.0 for Windows (SPSS Inc, Chicago, USA). Data were expressed as the mean, standard deviation (SD), median, interquartile range (IQR), percentage, sensitivity, specificity, positive predictive value (PPV), and negative predictive value (NPV). The chi-square test and Fisher’s exact test were used to compare categorical variables. An independent *t*-test was used to compare continuous variables, and the Mann-Whitney U test was used to compare nonparametric variables. The optimal cut-off levels for SHARP1 were determined from the receiver operating characteristic (ROC) curve. A p value of <0.05 was considered statistically significant.

## Results

Four hundred and thirteen women were enrolled in this study. Eight women were excluded: four carried fetuses with abnormalities, one had an abortion, one had an underlying disease of deep venous thrombosis with enoxaparin usage, and the other two were lost to follow-up. A total of 405 pregnant women were included in the analysis. Thirty-five women had preeclampsia (8.6%), and six of these had early-onset preeclampsia (1.5%).

Table 1 shows the demographic data, and the maternal and neonatal outcomes. There were no significant differences in age, parity, GA at measurement and total weight gain during pregnancy between preeclamptic and non-preeclamptic women. Preeclamptic women had a higher body mass index (BMI) than non-preeclamptic women. Preeclamptic women also had a lower GA at delivery and a higher preterm delivery rate than non-preeclamptic women.

**Table 1:** Demographic data, and maternal and neonatal outcomes of women with preeclampsia compared with women without preeclampsia.

	Women without preeclampsia (n = 370)	Women with preeclampsia (n = 35)	p value
Maternal age (years)	32.4 ± 4.1	32.3 ± 5.4	0.89
Nulliparous	224 (60.5)	25 (71.4)	0.21
GA at measurement (weeks)	12.6 ± 0.7	12.6 ± 0.8	0.86
BMI (kg/m^2^)	22.0 ± 3.5	24.2 ± 5.9	<0.01
Obesity	14 (3.8)	5 (14.3)	0.02
Total weight gain (kg)	14.0 ± 4.3	13.2 ± 5.8	0.30
GA at delivery (weeks)	38.5 ± 1.4	36.5 ± 2.8	<0.01
Delivery at GA < 37 weeks	20 (5.4)	15 (42.9)	<0.01
**Mode of delivery**			
Vaginal delivery	133 (35.9)	10 (28.6)	0.38
Cesarean delivery	230 (62.2)	25 (71.4)	0.27
Birthweight (grams)	3088.8 ± 404.7	2655.5 ± 710.0	<0.01
Fetal IUGR	5 (1.4)	3 (8.6)	0.03
**Apgar score**			
1 min < 7	4 (1.1)	3 (8.6)	<0.02
5 min < 7	1 (0.3)	2 (5.7)	<0.02
RDS	8 (2.2)	10 (29.4)	<0.01
Perinatal death	0	2 (5.7)	0.01

BMI: body mass index, GA: gestational age, IUGR: intrauterine growth restriction, RDS: respiratory distress syndrome.

Data are presented as the mean ± SD or as N (%).

Regarding neonatal outcomes, the fetuses of preeclamptic women had a lower neonatal birth weight than those of non-preeclamptic women. The fetuses of preeclamptic women also had higher rates of low Apgar scores, IUGR, respiratory distress syndrome (RDS) and perinatal death than those of pregnant women without preeclampsia.

### Serum SHARP1 levels and Doppler findings of uterine artery

Preeclamptic women had significantly lower serum SHARP1 levels than non-preeclamptic women (3.6 ng/ml vs 4.7 ng/ml, p < 0.01) (Table 2). There were no significant differences in the mean PI and the presence of an early diastolic notch between women with and without preeclampsia (p = 0.66 and 0.51, respectively).

**Table 2:** Serum SHARP1 level and uterine artery Doppler findings in women with preeclampsia and without preeclampsia.

	Women without preeclampsia (n = 370)	Women with preeclampsia (n = 35)	p value
SHARP1 (ng/ml)	4.7 ± 2.3	3.6 ± 1.3	< 0.01
UtA PI	1.60 (1.32, 1.97)	1.61 (1.40, 1.93)	0.66
Notching	201 (54.3)	17 (48.6)	0.51
Bilateral notching	121 (32.7)	10 (28.6)	0.62

Data are presented as the mean ± SD, median (IQR) or N (%).

UtA, uterine artery; PI, pulsatility index.

Women with early-onset preeclampsia and late-onset preeclampsia also had lower serum SHARP1 levels than non-preeclamptic women (3.3 ng/ml vs 4.7 ng/ml, p < 0.01 and 3.7 ng/ml vs 4.7 ng/ml, p < 0.01, respectively) (Table 3).

**Table 3:** Serum SHARP1 levels and uterine artery Doppler findings in women with early-onset and late-onset preeclampsia compared with women without preeclampsia.

	Women without preeclampsia (n = 370)	Women with early-onset preeclampsia (n =6)	Women with late-onset preeclampsia (n=29)	p value
SHARP1(ng/ml)	4.7 ± 2.3	3.3 ± 0.4		<0.01
			3.7 ± 1.5	<0.01
UtA PI	1.60 (1.3, 2.0)	1.72 (1.4, 2.8)		0.27
			1.61 (1.4, 1.8)	0.98
Notching	201 (54.3)	5(83.3)		0.23
			12 (41.4)	0.18
Bilateral notching	121 (32.7)	3 (50.0)		0.4
			7 (24.1)	0.34

Data are presented as the mean ± SD, median (IQR) or N (%).

UtA, uterine artery; PI, pulsatility index.

### Predictive value

**Figure 1:**
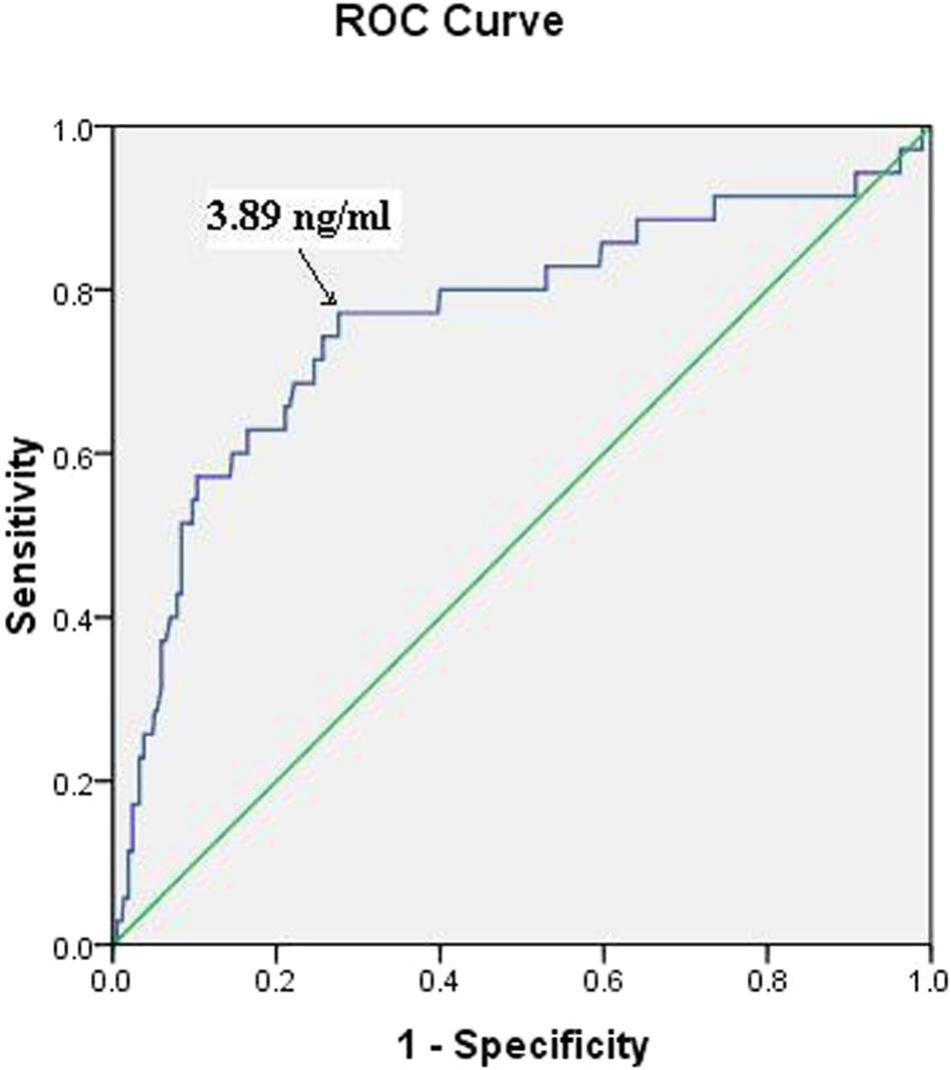
Receiver-operating characteristic curve for the relationship between the serum SHARP1 level and the diagnosis of preeclampsia. (area under the curve: 0.763; 95% confidence interval: 0.67–0.86; p < 0.01).

A receiver operating characteristic curve (ROC) was used to establish the cut-off value of serum SHARP1 levels, and the value was 3.89 ng/ml (AUC = 0.763, p < 0.01) (Fig. 1). The 95^th^ percentile for the mean PI was calculated from the study cohort. The 95^th^ percentile for the mean PI was determined for three groups according to GA. The 95^th^ percentiles for the mean PI were 2.83, 2.40 and 2.38 at 11–11^+6^, 12–12^+6^ and 13-13^+6^ weeks, respectively.

The sensitivity, specificity, PPV and NPV of serum SHARP1 levels less than 3.89 ng/ml for predicting preeclampsia were 77.1%, 72.7%, 21.1% and 97.1%, respectively. For uterine artery Doppler, the sensitivity, specificity, PPV and NPV of a mean PI > 95^th^ percentile for predicting women with preeclampsia were 5.7%, 95.4%, 10.5% and 91.5%, respectively. The sensitivity, specificity, PPV and NPV were 77.1%, 70.3%, 19.7% and 97.0%, respectively, for the combination of serum SHARP1 levels with a cutoff value of less than 3.89 ng/ml and a mean PI > 95^th^ percentile (Table 4).

**Table 4:** Predictive value of serum SHARP1 levels and uterine artery Doppler for preeclampsia.

	Sensitivity (%)	Specificity (%)	PPV (%)	NPV (%)	Positive LR	Negative LR
**Preeclampsia**						
SHARP1 < 3.89 ng/ml	77.1	72.7	21.1	97.1	2.8 (2.2, 3.6)	0.3 (0.2, 0.6)
UtA PI > 95^th^ percentile	5.7	95.4	10.5	91.5	1.2 (0.3, 5.2)	1.0 (0.9, 1.1)
Abnormal SHARP1 level and/or UtA PI	77.1	70.3	19.7	97.0	2.6 (2.0, 3.3)	0.3 (0.2, 0.6)
**Early-onset preeclampsia**						
SHARP1 < 3.89 ng/ml	100	72.7	5.6	100	3.6 (3.1, 4.3)	0
UtA PI > 95^th^ percentile	16.7	95.4	5.6	98.6	3.6 (0.6, 3.0)	0.9 (0.6, 1.3)
Abnormal SHARP1 level and/or UtA PI	100	70.3	5.2	100	3.4 (2.9, 3.9)	0

PPV: positive predictive value, NPV: negative predictive value, LR: likelihood ratio, UtA: uterine artery, PI: pulsatility index.

The positive LR and negative LR values are presented as time (95% confidence interval).

**Figure 2:**
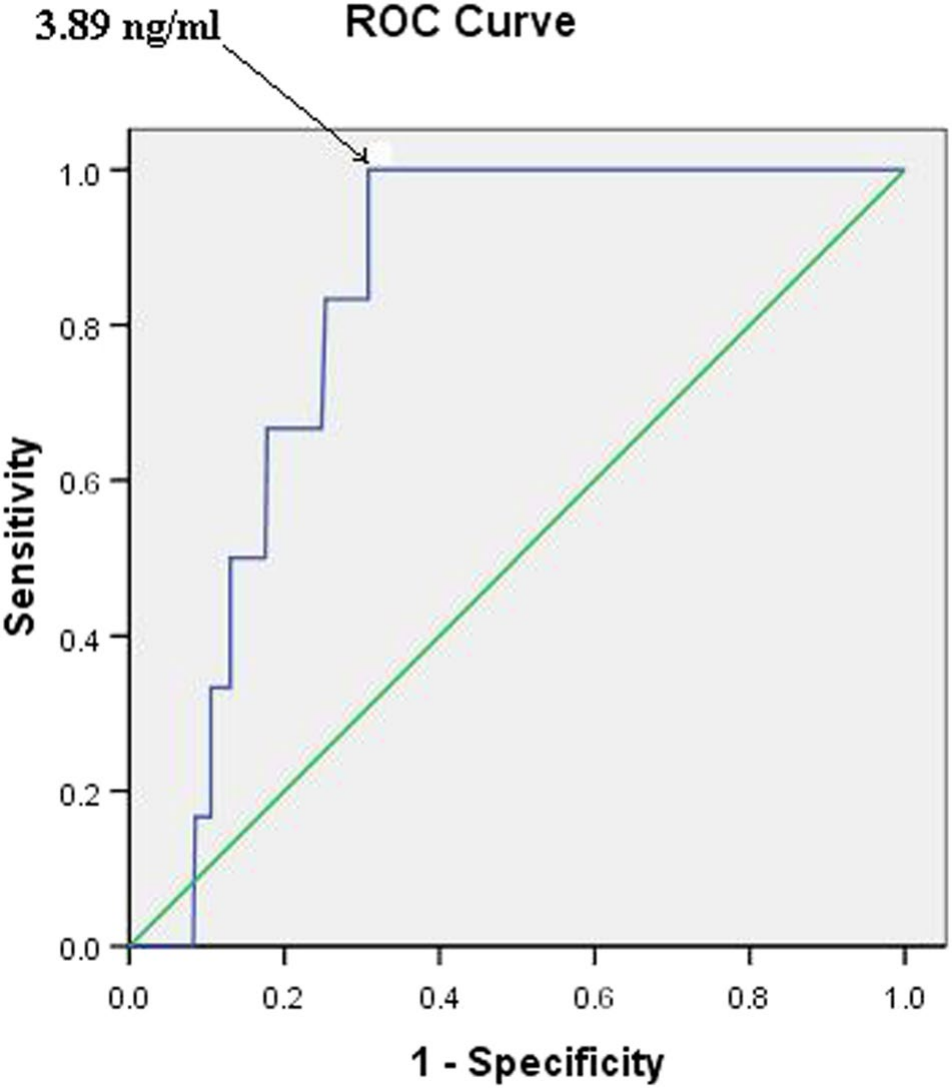
Receiver-operating characteristic curve for the relationship between the serum SHARP1 level and the diagnosis of early-onset preeclampsia. (area under the curve: 0.824; 95% confidence interval: 0.75–0.89; p = 0.003).

The sensitivity, specificity, PPV and NPV of abnormal serum SHARP1 levels for predicting early-onset preeclampsia were 100%, 72.7%, 5.6% and 100%, respectively (Table 4) (Fig. 2).

## Discussion

This study demonstrated that serum SHARP1 levels in the first trimester were effective for predicting preeclampsia.

The serum SHARP1 levels found in this study were significantly lower in preeclamptic women than in non-preeclamptic women. This result was consistent with that of Ersoy AO *et al*’s study^18^, which found that serum SHARP1 levels in the second and third trimesters were lower in women with preeclampsia and early-onset preeclampsia than in women without preeclampsia. However, the serum SHARP1 cut-off value in this study was different from that in Ersoy AO *et al*.’s study (26.765 ng/ml)^18^. This discrepancy might be due to the difference in the GA at measurement and the study population.

The uterine artery PI and the prevalence of a uterine artery diastolic notch in this study did not differ between women with and without preeclampsia. This result was similar to that of a previous study^22^ that compared first trimester uterine artery Doppler and biomarkers in women with and without preeclampsia. The findings in present study were similar to those of many previous studies^23,24^ that found that the first trimester PI of the uterine artery could not be used as a single predictor for preeclampsia.

The results of this study demonstrated that the serum SHARP1 level had good predictive value when used alone or in combination with Doppler of the uterine artery to predict both overall preeclampsia and early-onset preeclampsia in first trimester screening. Our results differed from those of previous studies that found that a combination of maternal serum markers with Doppler of uterine artery had poor sensitivity and specificity for predicting preeclampsia in the first trimester, and could only predict early-onset preeclampsia^22,23^.

The strength of this study was that it is the first study to evaluate serum SHARP1 levels in the first trimester for predicting preeclampsia and the study is a well characterized prospectively collected cohort. The limitation was that there were few cases of early-onset preeclampsia. Another limitation was the lack of data on blood pressure levels in the first trimester and previous history of preeclampsia, which are moderate to strong risk factors for predicting preeclampsia and the lack of replication cohort or statistical validation of the prediction rates. Further studies with larger sample sizes of patients with early-onset preeclampsia should be conducted.

## Conclusion

This study demonstrated that serum SHARP1 is a promising biomarker for predicting preeclampsia in the first trimester.

## References

[1] Assis TR, Viana FP, Rassi S (2008). Study on the major maternal risk factors in hypertensive syndromes. Arq Bras Cardiol.

[2] Khan KS, Wojdyla D, Say L, Gulmezoglu AM, Van Look PF (2006). WHO analysis of causes of maternal death: a systematic review. Lancet.

[3] Bellamy L, Casas JP, Hingorani AD, Williams DJ (2007). Pre-eclampsia and risk of cardiovascular disease and cancer in later life: systematic review and meta-analysis. BMJ.

[4] Duckitt K, Harrington D (2005). Risk factors for pre-eclampsia at antenatal booking: systematic review of controlled studies. BMJ.

[5] Huppertz B, Meiri H, Gizurarson S, Osol G, Sammar M (2013). Placental protein 13 (PP13): a new biological target shifting individualized risk assessment to personalized drug design combating pre-eclampsia. Hum Reprod Update.

[6] O’Gorman N, Nicolaides KH, Poon LCY (2016). The Use of Ultrasound and other Markers for Early Detection of Preeclampsia. Womens Health.

[7] Obstetric and Gynecology statistical report. Department of Obstetric and Gynecology, Faculty of Medicine, Chulalongkorn University (2011–2015).

[8] Lam C, Lim KH, Karumanchi SA (2005). Circulating angiogenic factors in the pathogenesis and prediction of preeclampsia. Hypertension.

[9] First-Trimester R (2015). Assessment for Early-Onset Preeclampsia. Obstet Gynecol.

[10] Onwudiwe N, Yu CKH, Poon LCY, Spiliopoulos I, Nicolaides KH (2008). Prediction of pre-eclampsia by a combination of maternal history, uterine artery Doppler and mean arterial pressure. Ultrasound Obstet Gynecol.

[11] Mikat B (2012). Early detection of maternal risk for preeclampsia. ISRN Obstet Gynecol.

[12] Kulmala L, Phupong V (2014). Combination of plasma-soluble fms-like tyrosine kinase 1 and uterine artery Doppler for the prediction of preeclampsia in cases of elderly gravida. Hypertens Res.

[13] Puttapitakpong P, Phupong V (2016). Combination of serum angiopoietin-2 and uterine artery Doppler for prediction of preeclampsia. Hypertens Res.

[14] Kato Y, Kawamoto T, Fujimoto K, Noshiro M (2014). DEC1/STRA13/SHARP2 and DEC2/SHARP1 Coordinate Physiological Processes, Including Circadian Rhythms in Response to Environmental Stimuli. Curr Top Dev Biol.

[15] Semenza GL (2001). Hypoxia-inducible factor 1: oxygen homeostasis and disease pathophysiology. Trends Mol Med.

[16] Sato F (2008). Basic-helix-loop-helix (bHLH) transcription factor DEC2 negatively regulates vascular endothelial growth factor expression. Genes Cells.

[17] Caniggia I (2000). Hypoxia-inducible factor-1 mediates the biological effects of oxygen on human trophoblast differentiation through TGF beta(3). J Clin Invest.

[18] Ersoy AO (2017). Maternal venous SHARP1 levels in preeclampsia. J Perinat Med.

[19] Roberts JM (2013). Hypertension in Pregnancy Report of the American College of Obstetricians and Gynecologists’ Task Force on Hypertension in Pregnancy. Obstet Gynecol.

[20] Aksornphusitaphong A, Phupong V (2013). Risk factors of early and late onset pre-eclampsia. J Obstet Gynaecol Res.

[21] Bhide A (2013). ISUOG Practice Guidelines: use of Doppler ultrasonography in obstetrics. Ultrasound Obstet Gynecol.

[22] Aksornphusitaphong A, Phupong V (2018). Combination of serum histidine-rich glycoprotein and uterine artery Doppler to predict preeclampsia. Hypertens Res.

[23] Odibo AO (2011). First-trimester placental protein 13, PAPP-A, uterine artery Doppler and maternal characteristics in the prediction of pre-eclampsia. Placenta.

[24] Schwartz N, Sammel MD, Leite R, Parry S (2014). First-trimester placental ultrasound and maternal serum markers as predictors of small-for-gestational-age infants. Am J Obstet Gynecol.

